# Five teacher profiles in student-centred curricula based on their conceptions of learning and teaching

**DOI:** 10.1186/1472-6920-14-220

**Published:** 2014-10-16

**Authors:** Johanna CG Jacobs, Scheltus J van Luijk, Francisca Galindo-Garre, Arno MM Muijtjens, Cees PM van der Vleuten, Gerda Croiset, Fedde Scheele

**Affiliations:** Department of Research in Education, VUmc School of Medical Sciences, P.O. Box 7057 (MF, A-114), 1007 MB Amsterdam, The Netherlands; LEARN! Research Institute for Learning and Education, VU University, Amsterdam, The Netherlands; Department of Resident Training, Maastricht University Medical Centre +, Maastricht, The Netherlands; Department of Biostatistics, VU University Medical Centre, Amsterdam, The Netherlands; Department of Educational Research and Development, Faculty of Health and Life Sciences and Medicine, Maastricht University, Maastricht, The Netherlands; Department of Obstetrics and Gynecology, St Lucas Andreas Hospital, Amsterdam, The Netherlands

**Keywords:** Conceptions of learning and teaching, Teachers, Reflection, Faculty development

## Abstract

**Background:**

Teachers’ conceptions of learning and teaching are partly unconscious. However, they are critical for the delivery of education and affect students’ learning outcomes. Lasting changes in teaching behaviour can only be realized if conceptions of teachers have been changed accordingly. Previously we constructed a questionnaire named COLT to measure conceptions. In the present study, we investigated if different teacher profiles could be assessed which are based on the teachers’ conceptions. These teacher profiles might have implications for individual teachers, for faculty development activities and for institutes. Our research questions were: (1) Can we identify teacher profiles based on the COLT? (2) If so, how are these teacher profiles associated with other teacher characteristics?

**Methods:**

The COLT questionnaire was sent electronically to all teachers in the first three years of the undergraduate curriculum of Medicine in two medical schools in the Netherlands with student-centred education. The COLT (18 items, 5 point Likert scales) comprises three scales: ‘teacher centredness’, ‘appreciation of active learning’ and ‘orientation to professional practice’. We also collected personal information about the participants and their occupational characteristics. Teacher profiles were studied using a K-means cluster analysis and calculating Chi squares.

**Results:**

The response rate was 49.4% (N = 319/646). A five-cluster solution fitted the data best, resulting in five teacher profiles based on their conceptions as measured by the COLT. We named the teacher profiles: Transmitters (most traditional), Organizers, Intermediates, Facilitators and Conceptual Change Agents (most modern). The teacher profiles differed from each other in personal and occupational characteristics.

**Conclusions:**

Based on teachers’ conceptions of learning and teaching, five teacher profiles were found in student-centred education. We offered suggestions how insight into these teacher profiles might be useful for individual teachers, for faculty development activities and for institutes and departments, especially if involved in a curriculum reform towards student-centred education.

## Background

A teacher’s personal views on learning and teaching are important in creating an inspiring learning environment for students [1, 2]. Previous research demonstrated that these views, or conceptions, are partly unconscious [3]. Conceptions of learning and teaching can be described as specific meanings attached to phenomena which act as filters through which new information passes as it is processed [4].

There is a difference between beliefs or conceptions of teachers, and teaching strategies or approaches. Beliefs and conceptions describe how teachers think about learning and teaching, while teaching strategies or approaches describe how they teach. Teaching strategies change more rapidly than conceptions depending on the demands of different teaching environments (lectures or small group teaching, first year or third year students) [5].

Several authors have argued that teachers’ conceptions influence their teaching approaches [1, 3, 6, 7] and some have demonstrated that teachers’ conceptions of learning and teaching indirectly affect students’ learning outcomes [2, 8, 9]. Furthermore, it is argued that changes in teaching behaviour can only be realized if attention is paid to teachers’ conceptions of learning and teaching [6, 10–12]. This often implies that faculty development activities should try to influence teachers’ conceptions to align them with the desired teaching behaviour [2, 13]. However, Guskey [14] stated that changes in teaching practice preceed changes in conceptions.

Contrary to other domains of higher education, medical education research has paid little attention to teachers’ conceptions of learning and teaching [15–17] and even less to its impact on faculty development activities [13]. To fill this gap, we constructed and validated a questionnaire named COLT, to measure teachers’ conceptions of learning and teaching in student-centred medical education [18]. As described by Harden et al. [19] ‘in student-centred education students have more responsibility towards their own learning (what and how) and in teacher-centred education learning is more passive, the teacher is the key figure, and there is an emphasis on lectures and laboratory work’. These concepts, however, are not polarized but represent a continuum.

In the current study, we explored teachers’ conceptions of learning and teaching more closely. We aimed to find out if profiles of teachers can be identified based on their conceptions.

Information on teacher profiles with different conceptions on learning and teaching might be interesting for several reasons. Firstly, it might be useful for teachers to know their partly unconscious conceptions, and insight into their teacher profiles might stimulate recognition. Next to reflection, it might even contribute to a change in conceptions [2]. Secondly, faculty development activities might also benefit from insights into the conceptions of participants. Thirdly, an organization-wide overview of teachers’ profiles, based on anonymous responses, might provide useful information if the institute is involved in a curriculum change towards student-centred education. Moreover, an organization-wide overview of teachers’ profiles might also be interesting if one realizes that teachers’ conceptions of learning and teaching are affected by their perceptions of the teaching context [20].

In other domains of higher education with conventional lecture-based curricula, categorizations of teachers have been reported before [1, 21–23]. In contrast to these studies, we specifically focused on student-centred medical education, concentrating on conceptions of teachers measured with a new validated questionnaire, and choosing a large-scale quantitative approach.

### Research questions

Our first research question was whether we can identify teacher profiles in student-centred medical education, based on the teachers’ scores on the COLT (with the scales ‘teacher centredness’ , ‘appreciation of active learning’ and ‘orientation to professional practice’). If so, the second research question was how these teacher profiles are associated with demographic and occupational characteristics of teachers.

We aimed to answer the first research question by conducting a cluster analysis, which groups similar teachers into subgroups. To answer the second question, we tested the associations with Chi square analyses.

## Methods

### Participants and setting

The COLT-questionnaire was sent to all teachers teaching in the first three years of the undergraduate curriculum of Medicine in two institutes with student-centred curricula in the Netherlands. These teachers were all involved in small group tutorials, lectures, practicals or skills training, or a combination of these. The two institutes were VU University Medical Centre in Amsterdam and Maastricht University Medical Centre, Maastricht, which will be referred to as Amsterdam and Maastricht. Amsterdam has had a student-centred curriculum for ten years, whereas Maastricht started with a student-centred curriculum when it was founded 40 years ago [24].

In both curricula, 8 to 12 students attend two sessions each week in which they tackle authentic problems. Teachers are expected to scaffold student learning rather than transmit knowledge. In Amsterdam, the learning objectives students work on are already given in the assignments, while in Maastricht, the problems are open and students generate the learning objectives themselves. Both curricula feature two to four lectures per week, as well as practicals and dedicated time for self-study. Basic sciences and clinical content are integrated in the learning material, and early patient contacts are included in both curricula.

### Ethical approval

The Ethical Review Committee of VUmc advised positively on the feasibility of this study. In the e-mail to the teachers we explicitly stated that participation was voluntary and that full anonymity was guaranteed.

### Instruments

To measure the teachers’ conceptions of learning and teaching, we used a validated questionnaire, the COLT (Conceptions on Learning and Teaching). Its construction, including validation and reliability testing, has been described before [18]. The questionnaire contains eighteen items (with five-point Likert scales: 1 = strongly disagree, 5 = strongly agree) that make up three independent scales: ‘teacher centredness’ , ‘appreciation of active learning’ and ‘orientation to professional practice’. The scale ‘teacher centredness’ contains eight items focusing on how important the respondent perceives his role as a teacher to be. In the list below we provide some examples of this scale. The scale ‘appreciation of active learning’ encompasses five items on the conceptions about active learning of students, in small group learning environments. Key components in this scale are how the teachers value students discussing the learning material, elaborating on and interpreting information instead of just absorbing knowledge [25]. The third COLT-scale ‘orientation to professional practice’ is composed of five items directed at the integration of future professional practice in the first three years of undergraduate medical education. This might be important to motivate students for deep learning activities and encapsulating theoretical knowledge for future practice (see below).

**Examples of the COLT (Conceptions of Learning and Teaching) - items**

*Factor 1: Teacher Centredness*

Students learn best when the learning process is guided by an expert who has an overview of the field of interest.

As a teacher I have to indicate clearly what is important and what is less important for the students to know.

I think that as an expert in my field I am eminently suitable to transmit my knowledge to students and that students should not have to look up that knowledge for themselves.

*Factor 2: Appreciation of Active Learning*

Students learn a great deal by explaining subject matter to each other.

Small group learning motivates students to study.

I think it is more important for students to be able to analyse and critically appraise subject matter than to memorise facts.

*Factor 3: Orientation to Professional Practice*

I think it is important that educational assignments are derived as much as possible from the students’ future professional practice.

Being introduced to the day-to-day practice of their future profession motivates students to learn.

It is a good learning outcome when students demonstrate that they can apply their knowledge during activities in situations in professional practice.

(Jacobs JCG, Van Luijk SJ, Van Berkel H, Van der Vleuten CPM, Croiset G, Scheele F: Development of an instrument (the COLT) to measure conceptions on learning and teaching of teachers, in student-centred medical education. *Med Teacher* 2012, 34:e483-e491; Available at http://colt.vumc.nl) Each item is rated on a five-point Likert scale (1 = strongly disagree, 5 = strongly agree).

We also collected additional information about the participants: medical school, gender, age (50 years or younger vs. older than 50 years), discipline (medical doctors, basic scientists or other), rank (associate professor/professor, or not), teaching experience (less than 5 years vs. 5 years or more), hours dedicated to education (less than 25% of total appointment vs. 25% or more of total appointment), educational role (delivery of education, or delivery combined with educational management, development and/or research) and type of educational task (only lectures, tutoring small groups, combined: lecturing and tutoring, and/or practicals or skills training).

### Procedure

A web-based version of the COLT and the second questionnaire was distributed among all teachers (N = 646) using Netquestionnaires™. Data were collected between November 2009 and April 2010. Non-responders received one e-mail reminder, followed by one reminder by telephone or letter. A small gift (booklet) was sent to the participating teachers. In the e-mail to the teachers we explicitly stated that participation was voluntary and that full anonymity was guaranteed.

### Analysis

For each respondent, we calculated the mean score for each of the three scales of the COLT, ‘teacher centredness’ , ‘appreciation of active learning’ , and ‘orientation to professional practice’. Questionnaire forms that were filled in incompletely (N = 13) were excluded from the analysis.

We used a cluster analysis as the technique to establish different teacher profiles, performed with SPSS version 19. With this technique objects are grouped or clustered into subgroups of objects that resemble each other more than they resemble other objects outside these subgroups. To identify profiles of teachers with different conceptions of learning and teaching, we used a K-means cluster analysis based on the three scales. In the clustering we tried fitting 2-cluster, 3-cluster, 4-cluster, 5-cluster and 6-cluster solutions according to the methods described for cluster analysis [26, 27]. As a validation procedure, we conducted a split half cross-validation procedure for the total group respondents as well as a cluster analysis for each medical school separately, followed by a split half cross-validation procedure for each medical school.

Subsequently, associations were explored between each teacher profile and each of the demographic and occupational characteristics of the teachers. A Chi-square test was used to assess statistical significance of the association, and p-values < 0.05 were considered significant.

## Results

The response rate of the COLT was 49.4%, since 319 of the 646 teachers filled in the questionnaire (for Amsterdam 49.6%, N = 184/371 and for Maastricht 49.1%, N = 135/275). Responders and non-responders were comparable on gender and discipline, both for the whole group and for the two medical schools separately.

In the cluster analysis, the five-cluster solution fitted the data best, based on meaningful differences and the distribution of teachers. We named the five teacher profiles ‘Transmitters’ , ‘Organizers’ , ‘Intermediates’ , ‘Facilitators’ and ‘Conceptual Change Agents’. Table 1 provides the descriptives of these profiles.

**Table 1:** **Descriptives of the five teacher profiles**

	Means (S.D.) (N = 319)	Teacher profiles				
		Transmitters (N = 19)	Organizers (N = 80)	Intermediates (N = 70)	Facilitators (N = 65)	Conceptual change agents (N = 85)
**Teacher centredness**	3.43 (.56)	4.06 (++)	3.81 (+)	3.76 (+)	3.25 (-)	2.78 (- -)
**Appreciation of active learning**	3.85 (.52)	2.72 (- -)	3.45 (-)	4.16 (+)	3.91 (=)	4.17 (+)
**Orientation to professional practice**	4.23 (.46)	3.44 (- -)	4.23 (=)	4.50 (+)	3.77 (- -)	4.54 (+)
***Medical schools:***						
**Maastricht (N,% over 5 profiles)**	135	6 (4.4%)	23 (17.0%)	24 (17.8%)	26 (19.3%)	56 (41.5%)
**Amsterdam (N,% over 5 profiles)**	184	13 (7.1%)	57(30.9%)	46 (25.0%)	39 (21.2%)	29 (15.8%)

The mean scores and standard deviations for the three scales ‘Teacher centredness’ , ‘Appreciation of Active Learning’ and ‘Orientation on future practice’ are provided for both the total group respondents (2nd column) and for the five teacher profiles. Also the distribution of the five profiles across the two medical schools is presented.

For ease of interpretation we added plus (+ or ++), minus (- or --) and (=) signs as a reference to the mean scores of the three COLT scales. A plus sign (+ or ++) indicates that for this teacher type the mean score is (much) higher than the overall mean score of all teachers on that scale, minus (- or --) refers to a score (much) lower than the overall mean score, and (=) signs refer to a score almost equal to the overall mean scores.

We decided to choose the scale ‘teacher centredness’ for ordering the five profiles, what resulted in a linear decrease in scores from the teacher profile Transmitters to the teacher profile Conceptual Change Agents. Because the three COLT scales are independent we did not expect a linear increase in the mean scores of the COLT scales ‘appreciation of active learning’ and ‘orientation to professional practice’. This was confirmed, due to a dip in mean scores for the ‘Facilitators’ profile (3.91 on ‘appreciation of active learning’; 3.77 for ‘orientation to professional practice’).

The teacher profiles ‘Transmitters’ and ‘Organizers’ can both be described as teacher-centred or more traditional, the ‘Intermediates’ have high scores on all scales and the ‘Facilitators’ and ‘Conceptual Change Agents’ are both low on ‘teacher centredness’ and high on ‘appreciation of active learning’.

‘Transmitters’ was the teacher type with the smallest number (N = 19) of teachers, comprising more teachers from Amsterdam (68.4%) than from Maastricht (31.6%). A metaphor corresponding to this teacher type is ‘the sage on the stage’ [28]. Most teachers from Maastricht (41.5%) fell into the ‘Conceptual Change Agent’ type, which can be labelled as having more modern conceptions, in line with student-centred education. A metaphor for this teacher type is ‘the guide by your side’ [28]. The distribution of the five teacher profiles in the two schools differed significantly (p <0.000).

As a validation procedure, we conducted a split half cross-validation procedure. This resulted in Cohen’s kappa of 0.067 and 0.033 (with p-values 0.030 and 0.349 respectively), which have to be interpreted as small. To explore the reason for this, we inspected a plot of the data, which revealed an overlap between the five clusters.

We also examined the cluster analysis for each medical school separately, followed by a split half cross-validation procedure for each medical school. This resulted in a five-cluster solution for Amsterdam and a two-cluster solution for Maastricht. For Amsterdam, the five clusters were comparable to the five profiles described before, whereas for Maastricht one cluster resembled the Transmitter profile and the other resembled the Conceptual Change Agents profile. The split half cross-validation performed for each medical school resulted in Cohen’s kappa of 0.253 and 0.254 for Amsterdam (both p = 0.000) and 0.253 and 0.359 for Maastricht (with p = 0.002 and 0.000 respectively). These kappa’s should be interpreted as small.

Next, we examined the five teacher profiles more closely by calculating Chi squares. The teacher profiles differed significantly in background and occupational characteristics, as is shown in Tables 2 and 3, e.g. in gender (p = 0.035) and in proportion and hours dedicated to education (p = 0.012). Teachers with less time for education were more often ‘Transmitters’ or ‘Organizers’.

**Table 2:** **Teacher profiles resulting from the cluster analysis of the COLT scales**

Teacher Profile		Demographic and occupational characteristics of the teacher profiles in the study sample
Name	COLT definition	
**Transmitters**	strong teacher centred conceptions (++)	More teachers from Amsterdam than from Maastricht, more female teachers, almost all dedicated less than 25% of their position to education, half of the teachers combine delivery of education with educational management, development and/or research, and about two-third of the teachers combine lectures with tutoring small groups and/or practicals or skills training, the other one-third of the teachers are only involved in tutoring small groups.
	very low appreciation of active learning (--)	
	very low orientation to future practice (--)	
**Organizers**	teacher centred conceptions (+)	More teachers from Amsterdam than from Maastricht, slightly more female teachers, most of them dedicated less than 25% of their position to education, slightly more teachers who combine delivery of education with educational management, development and/or research, and most of the teachers combine lectures with tutoring small groups and/or practicals or skills training.
	low appreciation of active learning (-)	
	average orientation to future practice (=)	
**Intermediates**	teacher centred conceptions (+)	Intermediate type. More teachers from Amsterdam than from Maastricht, half female teachers, most of them dedicated less than 25% of their position to education, half of the teachers combine delivery of education with educational management, development and/or research, and two-third of the teachers combine lectures with tutoring small groups and/or practicals or skills training.
	high appreciation of active learning (+)	
	high orientation to future practice (+)	
**Facilitators**	low on teacher centred conceptions (-)	More teachers from Amsterdam than from Maastricht, more female teachers, one-third dedicated more than 25% of their position to education, almost all combined delivery of education with educational management, development and/or research, and almost all combine lectures with tutoring small groups and/or practicals or skills training.
	average appreciation of active learning (=)	
	very low orientation to future practice (--)	
**Conceptual change agents**	very low on teacher centred conceptions (--)	More modern conceptions. Two-third are teachers from Maastricht, slightly more male teachers, one-third dedicated more than 25% of their position to education, many combined delivery of education with educational management, development and/or research, and almost all combined lectures with tutoring small groups and/or practicals or skills training.
	high appreciation of active learning (+)	
	high orientation to future practice (+)

**Table 3:** **Distribution of demographic and occupational characteristics within each teacher type**

	Total group (N = 319)	Transmitters (N = 19)	Organizers (N = 80)	Intermediates (N = 70)	Facilitators (N = 65)	Conceptual change agents (N = 85)
Medical School						
- Maastricht (N,%)	135	6 (31.6%)	23 (28.7%)	24 (34.3%)	26 (40.0%)	56 (65.9%)
- Amsterdam (N,%)	184	13 (68.4%)	57 (71.3%)	46 (65.7%)	39 (60.0%)	29 (34.1%)
Gender						
- Men	147	6 (31.6%)	36 (45.0%)	34 (48.6%)	22 (33.8%)	49 (57.6%)
- Women	172	13 (68.4%)	44 (55.0%)	36 (51.4%)	43 (66.2%)	36 (42.4%)
Age		n.s.	n.s.	n.s.	n.s.	n.s.
- ≤ 50 yrs	210					
- > 50 yrs	109					
Discipline		n.s.	n.s.	n.s.	n.s.	n.s.
- Medical doctors	158					
- Basic scientists	92					
- Other (psychologist, sociologist)	69					
Hours spent on educational tasks (percentage of total number of working hours)						
- < 25%	240	18 (94.7%)	66 (82.5%)	56 (80.0%)	43 (66.2%)	57 (67.1%)
- ≥ 25%	79	1 (5.3%)	14 (17.5%)	14 (20.0%)	22 (33.8%)	28 (32.9%)
Educational role						
- delivery of education	111	10 (52.6%)	35 (43.7%)	33 (47.1%)	12 (18.5%)	21 (24.7%)
- delivery combined with educational management, development, and/or research	208	9 (47.4%)	45 (56.3%)	37 (52.9%)	53 (81.5%)	64 (75.3%)
Type of educational tasks						
- lectures only	20	1 (5.3%)	9 (11.3%)	8 (11.4%)	2 (3.1%)	0 (0%)
- combination: lecturing and small groups, and/or practicals or skills training	239	13 (68.4%)	55 (68.7%)	46 (65.7%)	56 (86.1%)	69 (81.2%)
-small groups only	60	5 (26.3%)	16 (20.0%)	16 (22.9%)	7 (10.8%)	16 (18.8%)
Rank		n.s.	n.s.	n.s.	n.s.	n.s.
- (associate) professor	60					
- no (associate) professor	259					
Teaching experience		n.s.	n.s.	n.s.	n.s.	n.s.
- < 5 yrs	86					
- ≥ 5 yrs	233					

Percentages are reported per cell and per teacher type if significant when calculating Chi squares; n.s. means not significant.

Furthermore, teachers who combined the delivery of education with educational management, development and/or research were distributed differently over the five profiles than those involved only in the delivery of education (p < 0.000). If teachers combined the delivery of education with educational management, development or research, they were more often found in the profiles ‘Facilitators’ or ‘Conceptual Change Agents’. Finally, teachers conducting [1] only lectures, [2] a combination of lectures and small groups, or [3] small groups only were distributed differently over the five teacher profiles (p = 0.016).

## Discussion and conclusion

Our first research question was whether we could identify teacher profiles in student-centred medical education, based on their conceptions of learning and teaching as measured with the COLT (with the scales ‘teacher centredness’ , ‘appreciation of active learning’ and ‘orientation to professional practice’). We hypothesized that information on teacher profiles might be interesting as feedback for individual teachers, for faculty development activities, and for institutes involved in a curriculum reform.

A cluster analysis resulted in five teacher profiles, which were named ‘Transmitters’ , ‘Organizers’ , ‘Intermediates’ , ‘Facilitators’ and ‘Conceptual Change Agents’. Teachers with the profile ‘Transmitters’ have a preference for teacher-centred education, while ‘Facilitators’ and ‘Conceptual Change Agents’ prefer student-centred education formats. Teachers clustered in the profile ‘Intermediates’ demonstrate conceptions that fit in teacher-centred education as well as in student-centred education.

We chose to arrange the five profiles in the order of scores of the ‘teacher centredness’ scale, thus representing a linear decrease in scores from the teacher profile ‘Transmitters’ to the teacher profile ‘Conceptual Change Agents’. Beforehand we hypothesized not to find a linear increase (or decrease) in the other COLT scales, because these are independent. This was confirmed, mainly due to a dip in mean scores for the ‘Facilitators’ profile (3.91 on ‘appreciation of active learning’ and 3.77 for ‘orientation to professional practice’).

Apparently there is a substantial group of teachers, the ‘Facilitators’ , with a relatively low score on the COLT scale ‘teacher centredness’ combined with moderate scores on ‘appreciation of active learning’ and ‘orientation to professional practice’. An explanation for this might be that teachers with this profile are lower in ‘appreciation of active learning’ and ‘orientation to professional practice’ because of their background and occupational variables. As presented in Table 3 the teachers in this profile come mainly (60%) from Amsterdam and though not statistically significant, it might be that they are more often basic scientists with a lower orientation to the professional practice of doctors. Further it might be caused by reasons we did not include in this study, e.g. a major research task, a shortage of teaching skills or a shortage of knowledge about active learning.

We think that the ‘Intermediates’ and the ‘Facilitators’ profiles must be seen as transitional teacher types, which would imply that there is a development in teachers’ conceptions from ‘Transmitters’ towards ‘Conceptual Change Agents’ , as was suggested by Kember [1] and by Postareff et al. [20]. Further research is necessary to confirm this hypothesis.

Another explanation might be that the somewhat striking scores of the profile Facilitators are related to our population, possibly it is not heterogeneous enough. Replication of study design in a larger scale sample with more medical schools might give more clarity.

Our second research question was how these teacher profiles were associated with other teacher characteristics. We found significant differences between the five teacher profiles in gender, hours spent on educational tasks, educational role (delivery of education, or delivery combined with educational management, development and/or research) and type of educational tasks (lectures only, small groups only, or a combination: lectures and small groups, and/or practicals or skills training).

An interesting finding is that a greater involvement in education – by participation in educational management, in educational development initiatives and/or participating in educational research activities – is associated with more teachers with the profiles ‘Facilitators’ and ‘Conceptual Change Agents’. An explanation might be that especially teachers with these profiles are interested in these fields, or that participating in these fields stimulated a change in teachers’ conceptions. This will have to be investigated further.

Furthermore, the overall distribution of the profiles over the medical schools differed significantly. Maastricht had more teachers with the profile ‘Conceptual Change Agents’ than Amsterdam. The most logical explanation for this is the longer exposure to student-centred education in Maastricht compared to Amsterdam, but future research will have to confirm this. Another explanation might be that the teachers in Maastricht who did not want to engage in this type of teaching, opted out of teaching or went elsewhere. Since the curriculum in Amsterdam involves more guidance and direction from staff, it might have resulted in more corresponding profiles of teachers, i.e. more ‘Organizers’ and ‘Intermediates’ than ‘Conceptual Change Agents’.

Categorizations of teachers have been reported before, but those studies were performed in traditional curricula with a key role for lectures [1, 11, 21–23]. The categorizations – which often also comprised five categories – were based on interviews with a limited number of teachers [1], or on a questionnaire measuring *approaches to teaching*, the ATI [10] or on a combination of these [22, 23]. Pratt et al. [21] described five *perspectives on teaching*, thus combining beliefs, intentions, and actions of teachers. In contrast, we specifically focused on student-centred medical education and we used a new, validated questionnaire aimed at *conceptions* of teachers, COLT, in a large scale quantitative approach [18].

Below we discuss some practical implications which teacher profiles might have. We focus on the use of teacher profiles for individual teachers, for faculty development activities, and for institutes in a curriculum change.

### Individual teachers

The results of the COLT questionnaire provide teachers with information about their own teacher profile. We suppose that this feedback with a written description of the profile will be more meaningful than numerical scores on the three COLT scales. We expect that getting informed about one’s conceptions, which were partly unconscious before, will, in combination with reflection, contribute to the further professional development of teachers [7, 29–31]. The feedback might be used in several ways: in a portfolio for a teacher qualification, as an assignment preceding training, in a mentoring programme [32] or in a community of practice [33].

### Faculty development activities

More attention is needed for teachers’ conceptions of learning and teaching in order to achieve lasting changes in teaching behaviour [7, 10–12]. This also holds for optimizing the transfer of faculty development activities to daily practice [34]. In pedagogical courses, Postareff et al. [7] even recommend paying more attention to teachers’ conceptions than to teaching techniques.

We assume that if teachers are aware of their conceptions of learning and teaching, this might be a starting point in actually changing their conceptions. Subsequently, they might plan a discussion on the conceptions with a mentor or with peer teachers in a community of practice. For faculty developers, insight into teachers’ conceptions will be helpful in tailoring their activities to the conceptions and needs of teachers.

Interestingly, several efforts have been described to change conceptions of teachers, both long-term e.g. one year [13, 35–37] and short-term e.g. a two-hour workshop [28], or four three-hour sessions [2]. We think that the ultimate goal of these efforts should not be a homogeneous staff with similar conceptions. A diversity in teacher profiles will contribute to an inspiring learning environment for students [38–40]. Next to a teacher’s profile other qualities of teachers and students are important to stimulate deep learning and challenge students. Examples of these qualities are engagement of teachers in their work and their ability to inspire and motivate students. From this perspective a diversity of teacher qualities and teaching approaches might be important to appeal to students who have different learning styles. In addition, curriculum characteristics (e.g. early patient contacts) are important for engaging students in deeper learning activities.

It is important to realize that teaching conceptions and profiles can differ from the teaching approach a teacher demonstrates. Postareff et al. introduced the description ‘consonance or dissonance in the description of teaching’ [22]. For example, a teacher with a ‘Transmitter’ profile can guide students in a student-centred learning environment, but may experience a dissonance in the description of teaching. However, Prosser et al. [41] suggested that courses with consonance in teacher approaches, result in higher quality learning outcomes. In line with this, we propose to change teachers’ conceptions and teaching profiles – or to select teachers - to optimize the fit between teaching profile and format.

### Institutes

Insight into teacher profiles might also have practical implications for institutes. Firstly, if an institute aims for a curricular reform towards more student-centred education, it is advisable to take the organization-wide distribution of teacher profiles into account, since this may be helpful in the choices of implementation strategies and investments. Secondly, the organization-wide overview of teacher profiles is also relevant because institutes and curricula have an impact on teachers’ conceptions, since conceptions are influenced by the perception of the teaching context [20, 42, 43]. Key elements in the teaching context would be leadership style [44, 45], ownership of the curriculum [46], educational climate [47, 48], and the departmental teaching context [13, 46, 49–52].

### Strengths and limitations

This study introduces the profiling of teachers in student-centred education in the medical domain. The profiling is based on a cluster analysis of conceptions of learning and teaching, as measured with a validated and reliable questionnaire, in a large-scale sample of teachers. Some practical implications are discussed.

A limitation of our study is that we investigated two institutes with student-centred curricula in the Netherlands. Consequently, our findings cannot readily be generalized to other institutes and other countries. Secondly, the response rate on our electronic questionnaire was low, as 319 of 646 teachers (49.4%) filled in the questionnaire. However, responders and non-responders were comparable on gender and discipline, both for the whole group and for the two medical institutes separately. Thirdly, the validation of the cluster-analysis was somewhat disappointing, although the five-cluster solution was appropriate with respect to meaning and distribution. Presumably, a larger scale sample from more medical schools is needed to validate the five-cluster solution. Another explanation might be that the cluster-analysis technique is inappropriate to discriminate properly between the teacher profiles. This might imply that qualitative research methods are better equipped to capture reality.

### Implications for future research

We recommend in-depth interviews with teachers about their conceptions of learning and teaching and their perception of the teaching environment. In a longitudinal design, repeated measures combined with interviews might enable to study how static the teachers profiles are. Also, it might be interesting to compare several medical schools which are involved in a curriculum reform towards student-centred curricula. This might yield extra information on the validity of the COLT and the five teacher profiles. We recommend further research on the relation between teachers’ conceptions of learning and teaching and their actual teaching practices.

These studies might offer further insight into teachers’ conceptions of learning and teaching in student-centred curricula, including a sound theoretical model and practical implications. In line with Bleakly [53], we think that sociocultural models of learning will provide a better insight into teachers’ conceptions than individual models of experiential learning [54] or the reflective learning approach by Schön [55], because sociocultural models combine a teacher’s personal perspective and the teaching environment.

## Authors’ information

Johanna CG Jacobs, MD, MSc is a PhD-student and a staff member involved in Faculty Development at VUmc School of Medical Sciences, Amsterdam. She is also affiliated to LEARN! Research Institute for Learning and Education, VU University, Amsterdam.

Scheltus J van Luijk, MD, PhD, is an Assistant Professor in the department of Resident Education of Maastricht University Medical Centre +, Maastricht.

Francisca Galindo-Garre, PhD, is a Statistician in the Department of Biostatistics, VU University Medical Centre, Amsterdam.

Arno MM Muijtjens, PhD, is an Assistant Professor, Statistician and Methodologist in the Department of Educational Research and Development at the Faculty of Health, Medicine, and Life Sciences at Maastricht University, Maastricht.

Cees PM van der Vleuten, PhD, is a professor of Education, Chair of the Department of Educational Development and Research, and Scientific Director of the School of Health Professions Education (SHE) at Maastricht University, Maastricht.

Gerda Croiset, MD, PhD is a professor of Medical Education at the Vrije Universiteit Amsterdam, Director of VUmc School of Medical Sciences Amsterdam, and also affiliated to LEARN! Research Institute for Learning and Education, VU University, Amsterdam.

Fedde Scheele, MD, PhD, is a professor of Medical Education at the Vrije Universiteit Amsterdam and a gynaecologist at St. Lucas Andreas Hospital, Amsterdam.

## Abbreviations

ATI: Approaches to Teaching Inventory, constructed by Trigwell and Prosser
COLT: Conceptions of Learning and Teaching questionnaire (Jacobs et al. 2012).
